# Effectiveness of impedance parameters for muscle quality evaluation in healthy men

**DOI:** 10.1186/s12576-020-00780-z

**Published:** 2020-10-31

**Authors:** Hiroki Sato, Takao Nakamura, Toshimasa Kusuhara, Kobara Kenichi, Katsushi Kuniyasu, Takaki Kawashima, Kozo Hanayama

**Affiliations:** 1 Department of Radiological Technology, Graduate School of Health Sciences, Okayama University, 2-5-1, Shikata-cho, Kita-ku, Okayama, Okayama 700-8558 Japan; 2 Department of Rehabilitation Center, Kawasaki Medical School Hospital, 577, Matsushima, Kurashiki, Okayama 701-0192 Japan; 3 Department of Physical Therapist, Faculty of Rehabilitation, Kawasaki University of Medical Welfare, 288, Matsushima, Kurashiki, Okayama 701-0193 Japan; 4 Department of Physical Therapist, Kawasaki Junior College of Rehabilitation, 672, Matsushima, Kurashiki, Okayama 701-0192 Japan; 5 Department of Rehabilitation Medicine, Kawasaki Medical School, 577, Matsushima, Kurashiki, Okayama 701-0192 Japan

**Keywords:** Phase angle, Bioelectrical impedance analysis, Cole–Cole model, Muscle quality

## Abstract

We investigated the relationship between impedance parameters and skeletal muscle function in the lower extremities, as well as the effectiveness of impedance parameters in evaluating muscle quality. Lower extremity impedance of 19 healthy men (aged 23–31 years) measured using the direct segmental multi-frequency bioelectrical impedance analysis were arc-optimized using the Cole–Cole model, following which phase angle (PA), ${R}_{i}/{R}_{e}$, and *β* were estimated. Skeletal muscle function was assessed by muscle thickness, muscle intensity, and isometric knee extension force (IKEF). IKEF was positively correlated with PA (*r* = 0.58, *p* < 0.01) and *β* (*r* = 0.34, *p* < 0.05) was negatively correlated with ${R}_{i}/{R}_{e}$ (*r* = − 0.43, *p* < 0.01). Stepwise multiple regression analysis results revealed that PA, β, and ${R}_{i}/{R}_{e}$ were correlated with IKEF independently of muscle thickness. This study suggests that arc-optimized impedance parameters are effective for evaluating muscle quality and prediction of muscle strength.

## Introduction

Skeletal muscle function has been shown to be influenced by both quantitative factors (e.g., number of muscle fibers and cross-sectional area) and qualitative factors [1]. Qualitative factors include an increase in noncontractile tissue (e.g., fatty infiltration in skeletal muscle and myofascial degeneration) [2]. These are known to be caused by inactivity even in young people [3]. In recent years, skeletal muscle dysfunction has attracted attention not only for the decline of physical function, but also for the risk of developing lifestyle diseases and mortality after their occurence, and for the quality of life [4–6]. Methods for evaluating muscle quality include physiological tests and diagnostic imaging tests such as computed tomography (CT), magnetic resonance imaging (MRI), and ultrasonography (US). Of these tests, US is noninvasive and does not limit the measurement location or posture. This test can be conducted in various settings such as medical institutions and sports facilities [7, 8]. Muscle intensity (MI), which quantifies the extent of black and white areas from cross-sectional images of the skeletal muscle taken with US, reflects noncontractile tissue (e.g., increase in intramyocellular lipids and connective tissue). MI is therefore expected to be an effective index for evaluating muscle quality [9]. However, there have been several problems regarding the reproducibility and sensitivity of evaluation with MI, including (1) fluctuating numerical values depending on the measurement method and instrument settings, making comparison with other research data difficult, and (2) the rate of change in noncontractile tissue and luminance is not linear [10].

Indirect body composition evaluation, which estimates skeletal muscle mass and body fat mass using differences in tissue electrical conductivity and transmittance in bioelectrical impedance analysis (BIA) and dual-energy X-ray absorptiometry, has recently become widespread. BIA is a non-invasive measurement technique based on the electrophysiological properties of biological tissues. Muscle mass evaluation using BIA has been shown to be as highly accurate as that by conventional measurement methods [11]. Impedance parameters of the biological tissue can also be qualitative factors in skeletal muscle evaluation because they reflect the mass and uniformity of cells and the condition of cell membranes [12]. Particularly, phase angle (PA), which is the phase difference between current and voltage, is related not only to survival rate, nutritional status [13], and the occurrence of sarcopenia and frailty [14], but also to muscle strength [15], exercise tolerance [16], and physical activity level [17] in the field of geriatrics as well as to physical fitness and sports science. Thus, it has attracted attention as a means of qualitative assessment of muscle cell function, which was previously difficult to evaluate noninvasively.

Thus, although impedance parameters are attractive for muscle quality evaluation, when used as objective indicators, they require correction to accommodate for changes in frequency characteristics associated with skeletal muscle physiology and decline in anatomical function due to aging or disease. A decline in skeletal muscle function causes changes in the cell membrane resistance and an increase in the noncontractile tissue, which changes the central relaxation frequency ${(f}_{c})$ at which reactance reaches its maximum value [18]. Thus, optimizing the arc using the Cole–Cole model is recommended to compensate for these changes and measure the maximum PA of the target muscle [19]. Employing the Cole–Cole model, the PA (PA_cole_) is calculated using ${R}_{i}/{R}_{e}$ (known as the intracellular fluid resistance-to-extracellular fluid resistance ratio) and the beta parameter (*β*) as shown in Eqs. 1 and 2, respectively. PA_cole_ is an indicator of the structural perfection of skeletal muscle cells. ${R}_{i}/{R}_{e}$ refers to the balance between the intracellular fluid resistance and extracellular fluid resistance, and *β* refers to cell homogeneity [20]. Therefore, PA_cole_ is said to be an indicator of the structural completeness of skeletal muscle cells [21]. Previous studies have shown that intracellular and extracellular water contents estimated using Ri/Re of the lower extremity are useful for evaluating the skeletal muscle mass [22]. *β* is one of the performance indices of the capacitor in the equivalent circuit model, and is an index of the uniformity of the measured structure. It is quantified on a scale from 0 to 1. 0, indicating non-uniform and perfectly uniform tissue, respectively [23]. It has been shown that changes in myofiber type and fatty infiltration could be assessed using this parameter [24].

However, to the best of our knowledge, there has been no study on the relationship between muscle strength and muscle mass and arc-optimized impedance parameters (PA_cole_, ${R}_{i}/{R}_{e}$, *β*), focusing on the lower extremity skeletal muscle function. We presumed that PA_cole_ and ${R}_{i}/{R}_{e}$, *β* could be effective for the qualitative assessment of skeletal muscle composition related to muscle strength. This study aimed to verify the effectiveness of impedance parameters by simultaneously evaluating muscle thickness (MT) and MI with arc-optimized impedance parameters and US using Cole–Cole analysis for muscle quality evaluation to predict muscle strength.

## Methods

### Subjects

Nineteen healthy adult men (aged 23–31 years), with a total of 38 left and right lower extremities, were included. The study protocol was approved by the ethics review board of the Kawasaki Medical School (approval No.: 2846). Written informed consent was obtained from all participants. The inclusion criteria were as follows: (a) no history of lower extremity trauma or surgery; (b) no history of neuromuscular disorders; (c) not using an artificial pacemaker; (d) the ability to provide informed consent with no serious cognitive impairment, and (e) no regular exercise routine. Height and weight were measured, with the subjects standing barefoot and wearing light training clothes. Measurements were taken to the nearest 0.1 cm and 0.1 kg, and the body mass index (BMI) was calculated. Table 1 shows the physical characteristics of the participants.

**Table 1:** Physical characteristics and muscle srength, quantity and quality of the participants

Physical characteristics	Mean±SD
Age (year)	29.6±5.8
Height (cm)	172.4±4.3
Weight (kg)	68.8±10.7
BMI (kg/m^2^)	23.1±3.2
Isometric knee extension force (Nm)	180.8±31.8
Quadriceps femoris muscle thickness (mm)	86.0±9.5
Quadriceps femoris muscle intensity	86.9±19.3

*SD* standard deviation, *BMI* body mass index

### Experimental procedure

The impedance parameters were measured using direct segmental multi-frequency bioelectrical impedance analysis (DSM-BIA), MT, and MI were measured using US, and muscle strength was measured using isometric maximum muscle strength. The test procedures were uniform among all subjects. All assessments were performed on the same day. The subjects rested for 15 min in the supine position immediately before US and BIA measurements to stabilize body water [25].

### Bioelectrical impedance

Lower extremity impedance was measured with DSM-BIA using the InBody S10 (InBody Japan, Tokyo, Japan), which has a tetrapolar eight-point tactile electrode system and three different frequencies (5, 50, 250 kHz). Eight electrodes were attached to the thumb and middle finger of the hand for the upper limb, and to the back of the endocarpus and exocarpus for the lower limb. DSM-BIA measurements have been shown to be as accurate as those of the DEXA [11]. Measurements were taken in the supine position after 15 min of rest. The subjects were instructed to refrain from alcohol intake and excessive exercise on the day before the test. Measurements were performed in a controlled clinic room with a room temperature of 24–26 °C. Contact between trunk and extremities was prevented by placing the upper and lower extremities in the 30° abduction position [25].

#### Cole–Cole model

**Fig. 1:**
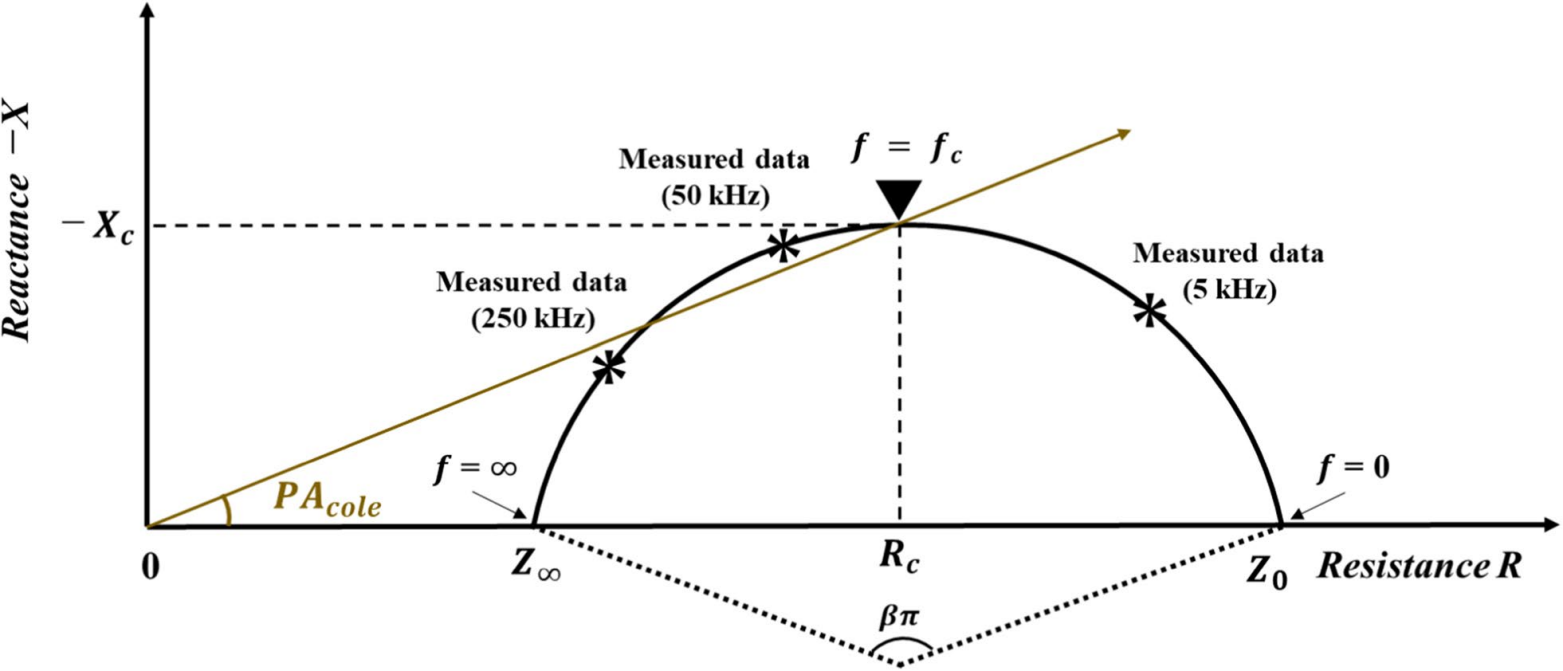
The arc of the Cole–Cole model

**Fig. 2:**
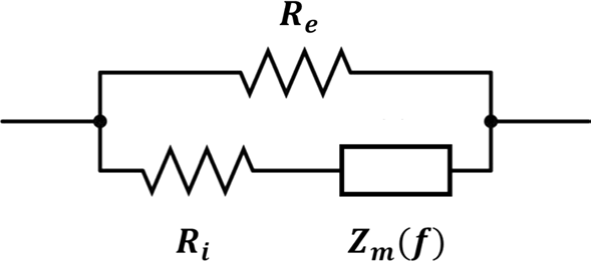
Equivalent circuit model of biological tissue

The Cole–Cole model was used to estimate the impedance parameters optimized for the arc [19]. The arc of the Cole–Cole model is shown in Eq. 1 and Fig. 1, and the biological equivalent circuit model is shown in Eq. 2 and Fig. 2:

1 $$Z\left( f \right) = R + jX = Z_{\infty } + \frac{{Z_{0} - Z_{\infty } }}{{1 + \left( {j\frac{f}{{f_{c} }}} \right)^{\beta } }},$$

2 $$\frac{1}{Z\left( f \right)} = \frac{1}{{R_{e} }} + \frac{1}{{R_{i} + Z_{m} }}\left( f \right),$$

where $f$ is the frequency, $Z(f)$ is the complex-valued impedance as a function of $f$, $R$ is the resistance, $X$ is the reactance, and *j* is an imaginary unit, ${Z}_{0}$ is $R$ when $f$ = 0, ${Z}_{0}$ is $R$ when $\mathrm{f}$ = $\infty$,  ${R}_{e}$ is the extracellular fluid resistance, ${R}_{i}$ is the intracellular fluid resistance, and ${Z}_{m}(f)$ is the cellular membrane impedance as a function of $f$. ${Z}_{\infty }$,${Z}_{0}, {f}_{c}, \beta$ were estimated using Eq. 1. $Z(f)$ and ${Z}_{m}(f)$ varies with $f$:

3 $$R_{e} = Z_{0} ,$$

4 $$R_{i} = \frac{{Z_{0} Z_{\infty } }}{{Z_{0} + Z_{\infty } }},$$

5 $$Z_{m} \left( f \right) = \frac{{\left( {Z_{0} } \right)^{2} }}{{Z_{0} - Z_{\infty } }}\left( {j\frac{f}{{f_{c} }}} \right)^{ - \beta } .$$

${R}_{\mathrm{c}}$ and $-{X}_{c}$ were determined from the obtained ${Z}_{0}$ and ${Z}_{\infty }$ and is given by

6 $$R_{{\text{c}}} = \frac{{Z_{0} + Z_{\infty } }}{2},$$

7 $$- X_{c} = \frac{{\left( {Z_{0} - Z_{\infty } } \right)\sin \frac{\beta \pi }{2}}}{{2\left( {1 + \cos \frac{\beta \pi }{2}} \right)}},$$

where ${R}_{c}$ is $R$ when the $f$ is ${f}_{c}$ and ${X}_{c}$ is $X$ when the $f$ is ${f}_{c}.$

${\mathrm{PA}}_{\mathrm{cole}}$ was measured from the obtained ${R}_{c}$ and $-{X}_{c}$ and is given by

8 $${\text{PA}}_{{{\text{cole}}}} = \arctan \left( {\frac{{ - X_{c} }}{{R_{c} }}} \right) = \frac{{R_{e} \sin \frac{\beta \pi }{2}}}{{\left( {R_{e} + 2R_{i} } \right)\left( {1 + \cos \frac{\beta \pi }{2}} \right)}} = \frac{{\sin \frac{\beta \pi }{2}}}{{\left( {1 + 2\frac{{R_{i} }}{{R_{e} }}} \right)\left( {1 + \cos \frac{\beta \pi }{2}} \right)}}.$$

The 50-kHz PA (PA_50_) used in previous research was added as a variable to evaluate the effects of the Cole–Cole analysis.

#### Search of $${{\varvec{Z}}}_{0}, {{\varvec{Z}}}_{\infty }{\varvec{\beta}},$$$${{\varvec{f}}}_{{\varvec{c}}}$$

The estimation of the four parameters was explored using an optimization method as shown in Eq. 9. Impedance and reactance data measured by DSM-BIA were used for the impedance data:

9 $$E = \mathop \sum \limits_{k = 1}^{3} \left. {\left\{ {\left( {R_{M} \left( {f_{k} } \right) - R_{E} \left( {f_{k} } \right)} \right)^{2} + \left( {X_{M} \left( {f_{k} } \right) - X_{E} \left( {f_{k} } \right)} \right)^{2} } \right.} \right\},$$

where ${f}_{1}$ = 5 kHz, ${f}_{2}$ = 50 kHz, ${f}_{3}$ = 250 kHz, ${R}_{M}\left({f}_{k}\right) and$ ${X}_{M}\left({f}_{k}\right)$ are the measured value of resistance and reactance at frequency ${f}_{k,}$ ${R}_{E}\left({f}_{k}\right) \mathrm\,and\,{X}_{E}\left({f}_{k}\right)$ are the resistance and reactance in Eq. 1 at the frequency ${f}_{k},$ respectively.

We searched for ${Z}_{0}, {Z}_{\infty }\beta$*,*${f}_{c}$ so that the evaluated value *E* was the minimum.

### Ultrasonography

**Fig. 3:**
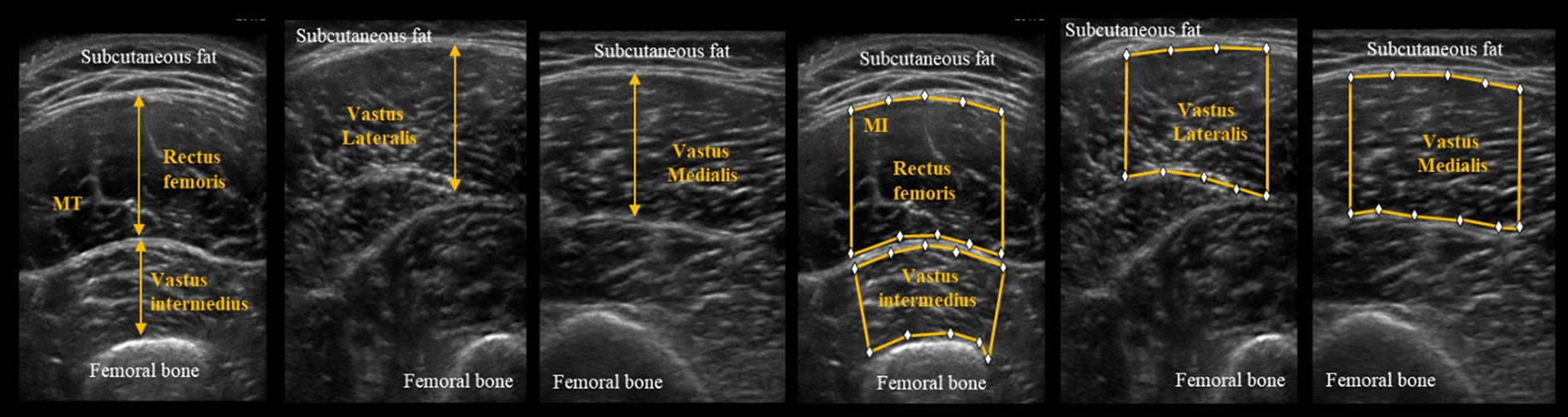
Measurement of muscle thickness (MT) and muscle intensity (MI)

Quadriceps femoris MT (QF_MT_) and MI (QF_MI_) were measured from cross-sectional images of the skeletal muscle using diagnostic ultrasound imaging equipment (SonoSite M-turbo, FUJIFILM). Measurements were taken in the brightness mode using a 6–15 MHz linear probe (56 mm). Hard-type echo gel (Conductor TM Transmission Gel, Chattanooga) was used to prevent the probe from touching the skin. Each measurement was taken twice. The target muscle group was the QF (rectus femoris, vastus medialis, vastus intermedius, and vastus lateralis). The measurement site for rectus femoris, vastus intermedius, and vastus lateralis was the midway point between the anterior superior iliac spine and the upper margin of patella, while that for vastus medialis was the point located 70% distal to the anterior superior iliac spine and the upper margin of the patella. Image analysis was performed using Image J-WinJP (LISIT, Tokyo, Japan) and using a partially modified method referencing the method proposed by Berger et al. [26]. Muscle luminance was measured using convex hull. After the upper and lower parts were surrounded to ensure the fascia was excluded, the values were quantified in the range of 0–255 with an 8-bit gray scale using histogram analysis, and the mean value of the area was calculated. QF_MT_ (mm) and QF_MI_ were calculated from the total value of the four muscles using the mean of two measurements taken for each muscle (Fig. 3). The measurements were taken by one examiner. The reliability of this measurement method has been demonstrated in previous studies [27].

### Muscle strength

Muscle strength was measured from isometric knee extension force (IKEF). The measurement was taken while the subject was seated in a chair without a backrest with the hip and knee joints at 90°. The equipment used for measurement was a handheld dynamometer (*μ*-TAS F-1, Anima, Japan). The sensor position was the distal part of the lower leg, and the arm length (*m*) was measured from the knee joint space to the center of the sensor. A 3-s maximum contraction was performed twice, with a 1-min break, and the maximum value (*N*) was used. The measured value was the value obtained by multiplying the measured value by the arm length (Nm).

### Statistical analysis

All data are expressed as mean ± standard deviation. All statistical analyses were performed in EZR (Ver 1.4, Saitama Medical Center, Jichi Medical University, Saitama, Japan) [28]. Significance level was set as less than 5% (*p* < 0.05). Pearson's correlation coefficient was used for calculating the correlation of IKEF with QF_MT_, QF_MI_, PA_50_, PA_cole_, *β*, ${R}_{i}/{R}_{e}$, ${f}_{c}$, and BMI. Stepwise multiple regression analysis was conducted with IKEF as the dependent variable to examine the effects of PA_cole_ and *β*, ${R}_{i}/{R}_{e}$ on IKEF. The linear models were as follows: Model 1, with QF_MT_, QF_MI_, and BMI as independent variables; Model 2, with PA_cole_ added; and Model 3, with *β* and ${R}_{i}/{R}_{e}$ added. Variance inflation factor (VIF) was calculated to confirm the existence of multicollinearity.

## Results

The subjects’ physical characteristics and data of IKEF, QF_MT_, QF_MI_, and lower extremity impedance parameters are given in Tables 1 and 2, respectively. Data of the correlation between the subjects’ IKEF and QF_MT_, QF_MI_, and lower extremity impedance parameters are shown in Table 3. IKEF had a significantly positive correlation with MT (*r* = 0.58, *p* < 0.01), PA_cole_ (*r* = 0.53, *p* < 0.01), and *β* (*r* = 0.55, *p* = 0.04) and a significantly negative correlation with ${R}_{i}/{R}_{e}$ (*r* = − 0.42, *p* < 0.01). The results of stepwise multiple regression analysis with IKEF as the objective variable are given in Table 4. QF_MT_ was shown to be significantly related in Model 1. PA_cole_, and *β* and ${R}_{i}/{R}_{e}$ were selected as significant variables in Models 2 and 3, respectively. The VIF was within the range of 1.05–1.36 for all variables, indicating no multicollinearity.

**Table 2:** Impedance parameter of the lower extremity

	Mean±SD
${R}_{c}$(Ω)	226.9±23.5
${X}_{c}$(Ω)	28.7±3.9
${Z}_{0}$(Ω)	274.4±28.4
${ Z}_{\infty }$(Ω)	181.6±18.4
${ f}_{c}$ (kHz)	38.1±8.1
${ R}_{i}/{R}_{e}$	1.98±0.23
$\upbeta$	0.71±0.03
PA_cole_ (deg)	7.17±0.52
PA_50_ (deg)	6.88±0.65

*SD *standard deviation, ${R}_{c}$ resistance of $f={f}_{c},$ ${X}_{c}$ reactance of $f={f}_{c},$ ${Z}_{0} \mathrm\,{resistance\,of }\,f=0,$ ${Z}_{\infty } \mathrm\,{resistance\,of }\,f=\infty$, ${f}_{c}$ central relaxation frequency, ${R}_{i}$/${R}_{e}$ ratio of intracellular fluid resistance to extracellular fluid resistance, $\beta$ beta parameter, *PA*_*cole*_ phase angle of Cole–Cole model, *PA*_*50*_ phase angle of 50 kHz

**Table 3:** Correlation coefficients between muscle strength, muscle strength, muscle thickness, muscle intensity, impedance parameters and physical characteristics of the lower extremities (*n*=38)

	IKEF	QF_MT_	QF_MI_	PA_cole_	PA_50_	$\beta$	${R}_{i}$/${R}_{e}$	${f}_{c}$	BMI
IKEF	–	0.578**	− 0.229	0.583**	0.105	0.34*	− 0.43**	− 0.027	0.032
QF_MT_		–	− 0.272	0.215	0.282	0.187	− 0.101	− 0.094	0.151
QF_MI_			–	− 0.05	− 0.295	0.122	0.124	− 0.056	− 0.305
PA_cole_				–	− 0.053	0.008	− 0.852**	− 0.29	0.079
PA_50_					–	− 0.015	0.003	− 0.068	0.187
$\beta$						–	0.554**	− 0.109	− 0.165
${R}_{i}$/${R}_{e}$							–	0.238	− 0.238
${f}_{c}$								–	− 0.343*
BMI									–

*IKEF *isometric knee extension force, *QF*_*MT*_ muscle thickness of quadriceps femoris, *QF*_*MI*_ muscle intensity of quadriceps femoris, *PA*_*cole*_ phase angle of Cole–Cole model, *PA*_*50*_ phase angle of 50 kHz, $\beta$ Beta parameter, ${R}_{i}$/${R}_{e}$ ratio of intracellular fluid resistance to extracellular fluid resistance, ${f}_{c}$ central relaxation frequency, *BMI* body mass index

Statistical significance: *p˂0.05, **p˂0.01

**Table 4:** Predictors of muscle strength in lower extremity (*n*=38)

Dependent variables	Independent variables	Coefficient	Standardized coefficient	*p* value	95% CI	VIF
Model 1						
${R}^{2}=$ 0.29	QF_MT_	19.13	0.57	<0.01	[9.26, 28.9]	1.09
	QF_MI_	− 0.16	− 0.09	0.53	[− 0.66, 0.34]	1.18
	BMI	− 0.89	− 0.09	0.55	[− 3.92, 2.13]	1.11
Model 2						
${R}^{2}=$ 0.53	QF_MT_	15.7	0.46	<0.01	[0.22, 0.71]	1.14
	QF_MI_	− 0.18	− 0.1	0.39	[− 0.35, 0.14]	1.18
	PA_cole_	29.8	0.49	<0.01	[0.25, 0.72]	1.05
	BMI	− 1.16	− 0.11	0.34	[− 0.35, 0.13]	1.11
Model 3						
${R}^{2}=$ 0.66	QF_MT_	11.6	0.35	<0.01	[0.13, 0.56]	1.23
	QF_MI_	− 0.15	− 0.09	0.37	[− 0.3, 0.12]	1.21
	${R}_{i}$/${R}_{e}$	− 79.2	− 0.43	<0.01	[− 0.64, -0.22]	1.14
	β	17.6	0.55	<0.01	[0.34, 0.76]	1.13
	BMI	− 0.95	− 0.09	0.36	[− 0.29, 0.11]	1.11

*R*^*2*^ represents the adjusted coefficient of determination

*CI *confidence intervals, *VIF* variance inflation factor, *QF*_*MT*_ muscle thickness of quadriceps femoris, *QF*_*MI*_ muscle intensity of quadriceps femoris, *PA*_*cole*_ phase angle of Cole–Cole model, $\beta$ beta parameter, ${R}_{i}$/${R}_{e}$ ratio of intracellular fluid resistance to extracellular fluid resistance, *BMI* body mass index

## Discussion

To the best of our knowledge, this is the first study to investigate the relationship between the two components (${R}_{i}/{R}_{e}$, *β*) of the phase angle in the central frequency from the Cole–Cole model of site-specific impedances of the lower extremity, using optimization calculations and conventional assessment of skeletal muscle function. Previous studies have reported the relationship between whole-body PA_50_ and upper and lower extremity muscle strength and exercise tolerance [15, 16]. In this study, we demonstrated the correlation between lower extremity impedance parameters (PA_cole,_ ${R}_{i}/{R}_{e}$, *β*, and ${f}_{c}$) and Q*F*_*M*T_, QF_MI_, IKEF, and BMI. Further, when PA_cole_ (Model 2) and ${R}_{i}/{R}_{e}$ and *β* (Model 3) were added as independent variables in the stepwise multiple regression analysis where IKEF was set as a dependent variable, it resulted in increased *R*^2^ and decreased QF_MT_ standardized partial regression coefficient (SC). A noteworthy finding is that in Model 3, both ${R}_{i}/{R}_{e}$ and *β* were shown to be factors that affect IKEF independent of QF_MT_. These results support our hypothesis that high muscle thickness and high PA_cole_ are independently associated with IKEF in healthy men and that impedance parameters (PA_cole_, ${R}_{i}/{R}_{e}$, *β*) as a muscle quality evaluation parameter enhances the suitability of muscle strength evaluation. This study suggests that simultaneous evaluation of QF_MT_ and impedance parameters (PA_cole_, ${R}_{i}/{R}_{e}$, *β*) could enable accurate estimation of muscle strength.

First, this study demonstrated the correlation between lower extremity impedance parameters (PA_cole,_ ${R}_{i}/{R}_{e}$, *β*, and ${f}_{c}$) and QF_MT_, QF_MI_, IKEF, and BMI (Table 3). Evaluation of skeletal muscle composition is important for predicting skeletal muscle function and physical function and for evaluating the effects of aging and disease [29]. PA_50_ has been shown to be related to muscle strength, exercise tolerance, fall history, and physical activity in the field of geriatrics, physical fitness, and sports science [15–17]. Furthermore, studies investigating changes before and after resistance training and age-related changes show that fluctuations in resistance and reactance associated with changes in skeletal muscle function affect PA_50_ [30]. Thus, although there are an increasing number of studies showing the relationship of skeletal muscle and physical function with impedance parameters, those studies use an impedance parameter with a frequency of only 50 kHz. Conventionally, the ${f}_{c}$ in biological tissues is approximated as 50 kHz, which is the reason for using the 50 kHz impedance value usually [31]. However, the change in muscle fiber size, increase in connective tissue, and fatty infiltration in skeletal muscle change the frequency characteristics of the skeletal muscle; therefore, the impedance parameter in ${f}_{c}$ using the Cole–Cole model is optimal for evaluating changes in skeletal muscle function [32]. In this study, the ${f}_{c}$ (38.1 ± 8.1 kHz) was lower than 50 kHz in all subjects. The results of this study showed a moderately positive correlation between IKEF and PA_cole_ (*r *= 0.58 *p* < 0.01), although PA_50_ did not show a significant correlation (*r* = 0.11, *p* = 0.53). Furthermore, there was a positive correlation between IKEF and *β* (r = 0.34, p = 0.04), and a moderately negative correlation with ${R}_{i}/{R}_{e}$ (*r* = − 0.43, *p *< 0.01). A notable finding is that ${f}_{c}$ did not have a significant correlation with muscle strength (*r* = − 0.03, *p* = 0.8). Previous studies indicated significant correlations between, ${R}_{i}/{R}_{e}$ and/or ${f}_{c}$, which constitute PA_cole_. However, there was no correlation with muscle strength and muscle mass [22, 33–36]. Most of these studies focused on age-related changes in the aging process in older adults and aged mice, focusing on changes in the intracellular water to extracellular water ratio and the decrease in phase angle associated with age-related muscle mass loss [22, 33–36]. Conversely, as shown in Eq. 8 and Fig. 1, in addition to the intracellular water to extracellular water ratio (${R}_{i}/{R}_{e}$ in the present study), the *β* has an effect on the phase angle. Despite the fact that *β* reflects cell homogeneity and may be affected by qualitative changes in skeletal muscle [20], no studies have focused on *β*. In the present study, *β* demonstrated a moderately positive correlation with IKEF, indicating that in addition to ${R}_{i}/{R}_{e}$, *β* is a factor that reflects the influence of skeletal muscle function.

Second, when PA_cole_ (Model 2) and ${R}_{i}/{R}_{e}$ and *β* (Model 3) were added as independent variables in stepwise multiple regression analysis with IKEF as a dependent variable, it resulted in increased *R*^2^ and decreased QF_MT_ SC in both models (Table 4). It has previously been reported that the prediction accuracy of muscle strength dramatically improves with a combination of muscle mass and muscle quality, rather than using muscle mass alone [37]. Additionally, the importance of evaluating muscle quality is increasing because the decline in muscle quality precedes the decline in muscle strength [38]. With the change from Models 1 to 2, the QF_MT_ SC decreased from 0.57 to 0.46, while *R*^2^ increased from 0.29 to 0.53. The PA_cole_ SC was 0.49, which was approximately the same value as that for QF_MT_, indicating that it had an effect independent of other variables. As expected, the analysis for predicting muscle strength produced results similar to those of the study conducted by Bourgeois et al. [37], where muscle mass and PA were set as variables. In Model 3, the components of PA_cole_ were divided into ${R}_{i}/{R}_{e}$ and *β*, and the effects of those variables were analyzed. New findings in this study indicate that both ${R}_{i}/{R}_{e}$ (SC: − 0.43, p < 0.01) and *β* (SC: 0.55, p < 0.01) are factors that affect IKEF independently of QF_MT_. The low contribution of QF_MI_ is due to the muscle quality evaluation with MI that tends not to show up as changes in the degree of whiteness unless there is more than a certain level of fascia degeneration or adipose tissue, which suggests that these changes may be underestimated in certain subjects [10]. In addition, as there is a difference in the percentage of fat infiltration between the distal part and the proximal part in the lower extremity [10], measurement accuracy can be improved by evaluating not just a single slice using US but evaluating a wide range. These findings suggest that using the impedance parameter in a wide range of tissues with DSM-BIA may enable more accurate detection of muscle quality condition than echo intensity (EI). The numerical values of ${R}_{i}/{R}_{e}$ and *β* in the evaluation of muscle quality may fluctuate due to various muscle quality disorders such as an increase in the noncontractile tissue, muscle fiber atrophy, and a decline in fascia function. Thus, further study is needed, analyzing each element separately. Our results show that ${R}_{i}/{R}_{e}$ and *β* can be used to evaluate muscle quality, which is difficult to express with EI, and that both are factors that independently affect muscle strength.

Evaluation of body composition using DSM-BIA is currently used in various fields such as medical care and athletes [39, 40]. Unlike CT and MRI, it does not require a special environment, and it is highly reproducible, making it widely used for training and evaluation of disease-related skeletal muscle mass loss. Evaluating muscle quality using a combination of PA_cole_, ${R}_{i}/{R}_{e}$, and *β*, rather than measuring skeletal muscle mass alone, may mean that skeletal muscle function can be predicted with higher accuracy than before. Prediction of skeletal muscle function using a combination of muscle mass and impedance parameters could be applied to various fields such as medical care, sports, and community-dwelling elderly once data have been accumulated considering race, gender, and age.

This study has several limitations. First, the subjects in this study were limited to healthy adult men. Given that gender, age, and nutritional status have an influence on the impedance parameters, the findings may not be applicable to other populations including women, the elderly, and sick patients. Second, it is necessary to consider that lower extremity impedance parameters in DSM-BIA may be affected by tissue impedance other than skeletal muscle. Furthermore, it has been shown that the 95% limits of agreement are larger than that of dual-energy X-ray absorptiometry and dilution-measured total body water methods [41]. In cases where it is possible to establish a measurement method that minimizes the effect of the skin and subcutaneous fat using electrical impedance myography applying BIA, it would be possible to improve the evaluation accuracy methods using skeletal muscle alone. Finally, this study did not perform physiological and anatomical evaluations showing evidence that PA_cole_, ${R}_{i}/{R}_{e}$, and *β* reflect the quality of the skeletal muscle.

Based on the results of this study, further research analyzing ${R}_{i}/{R}_{e}$ and *β* is required to increase the effectiveness of impedance parameters for evaluating muscle quality.

## Conclusion

This study shows that PA_cole_, ${R}_{i}/{R}_{e}$, and *β* calculated using the Cole–Cole model are factors that independently affect muscle strength, even when muscle mass is added to the variables. Thus far, this is the first study showing an association between the lower extremity impedance parameters in DSM-BIA and skeletal muscle function. These factors can be measured noninvasively in a short period of time, making them effective as muscle quality indicators in a wide range of subjects. ${R}_{i}/{R}_{e}$ and *β* may present with more characteristic changes than the results of this study in the elderly and in patients with diseases, although these points need further investigation.

## Data Availability

The datasets used and/or analyzed during the current study are available from the corresponding author on reasonable request.

## Abbreviations

*β*: Beta parameter (cell homogeneity)
BIA: Bioelectrical impedance analysis
BMI: Body mass index
CT: Computed tomography
DSM-BIA: Direct segmental multi-frequency bioelectrical impedance analysis
*f*_*c*_: Central relaxation frequency
MI: Muscle intensity
MRI: Magnetic resonance imaging
MT: Muscle thickness
PA: Phase angle
PA_cole_: Phase angle of Cole–Cole model
PA_50_: Phase angle of 50 kHz
QF_MT_: Muscle thickness of quadriceps femoris
QF_MI_: Muscle intensity of quadriceps femoris
*R*_*i*_*/R*_*e*_: Ratio of intracellular fluid resistance to extracellular fluid resistance
US: Ultrasonography
VIF: Variance inflation factor

## References

[1] Deschenes MR (2004). Effects of aging on muscle fibre type and size. Sports Med.

[2] Correa-de-Araujo R, Harris-Love MO, Miljkovic I, Fragala MS, Anthony BW, Manini TM (2017). The need for standardized assessment of muscle quality in skeletal muscle function deficit and other aging-related muscle dysfunctions: a symposium report. Front Physiol.

[3] Manini TM, Clark BC, Nalls MA, Goodpaster BH, Ploutz-Snyder LL, Harris TB (2007). Reduced physical activity increases intermuscular adipose tissue in healthy young adults. Am J Clin Nutr.

[4] Cruz-Jentoft AJ, Bahat G, Bauer J, Boirie Y, Bruyère O, Cederholm T, Cooper C, Landi F, Rolland Y, Sayer AA, Schneider SM, Sieber CC, Topinkova E, Vandewoude M, Visser M, Zamboni M, Writing Group for the European Working Group on Sarcopenia in Older People 2 (EWGSOP2), and the Extended Group for EWGSOP2 (2019). Sarcopenia: revised European consensus on definition and diagnosis. Age Ageing.

[5] Brown JC, Harhay MO, Harhay MN (2016). Sarcopenia and mortality among a population-based sample of community-dwelling older adults. J Cachexia Sarcopenia Muscle.

[6] Sayer AA, Syddall HE, Martin HJ, Dennison EM, Roberts HC, Cooper C (2006). Is grip strength associated with health related quality of life? Findings from the Hertfordshire Cohort Study. Age Ageing.

[7] Ticinesi A, Meschi T, Narici MV, Lauretani F, Maggio M (2017). Muscle ultrasound and sarcopenia in older individuals: a clinical perspective. J Am Med Dir Assoc.

[8] Loizides A, Gruber H, Peer S, Plaikner M (2017). Muscular injuries of athletes: Importance of ultrasound. Radiologe.

[9] Mayans D, Cartwright MS, Walker FO (2012). Neuromuscular ultrasonography: quantifying muscle and nerve measurements. Phys Med Rehabil Clin N Am.

[10] Young H-J, Jenkins NT, Zhao Q, McCully KK (2015). Measurement of Intramuscular Fat by Muscle Echo Intensity. Muscle Nerve.

[11] Ling CHY, de Craen AJM, Slagboom PE, Gunn DA, Stokkel MPM, Westendrop RGJ, Maier AB (2011). Accuracy of direct segmental multi-frequency bioimpedance analysis in the assessment of total body and segmental body composition in middle-aged adult population. Clin Nutr.

[12] Foster KR, Lukaski HC (1996). Whole-body impedance—what does it measure?. Am J Clin Nutr.

[13] Mullie L, Obrand A, Bendayan M, Trnkus A, Ouimet MC, Moss E, Chen-Tournoux A, Rudski LG, Afilalo J (2018). Phase Angle as a Biomarker for Frailty and Postoperative Mortality: The BICS Study. J Am Heart Assoc.

[14] Kilic MK, Kizilarslanoglu MC, Arik G, Bolayir B, Kara O, Dogan Varan H, Sumer F, Kuyumcu ME, Halil M, Ulger Z (2017). Association of bioelectrical impedance analysis-derived phase angle and sarcopenia in older adults. Nutr Clin Pract.

[15] Hetherington-Rauth M, Baptista F, Sardinha LB (2019). BIA-assessed cellular hydration and muscle performance in youth, adults, and older adults. Clin Nutr.

[16] Stahn A, Strobel G, Terblanche E (2008). VO2max prediction from multi-frequency bioelectrical impedance analysis. Physiol Meas.

[17] Uemura K, Yamada M, Saho K (2019). Association of bio-impedance phase angle and physical activity level in older adult. Jpn Phys Ther Assoc.

[18] Arnold WD, Taylor RS, Li J, Nagy JA, Sanchez B, Rutkove SB (2017). Electrical impedance myography detects age-related muscle change in mice. PLoS ONE.

[19] Cole KS, Cole RH (1941). Dispersion and absorption in dielectrics I. Alternating current characteristics. J Chem Phys.

[20] Sanchez B, Rutkove SB (2017). Electrical impedance myography and its applications in neuromuscular disorders. Neurotherapeutics.

[21] Barbosa-Silva MC, Barros AJ, Wang J, Heymsfield SB, Pierson RN (2005). Bioelectrical impedance analysis: population reference values for phase angle by age and sex. Am J Clin Nutr.

[22] Yamada Y, Buehring B, Krueger D, Anderson RM, Schoeller DA, Binklet N (2016). Electrical properties assessed by bioelectrical impedance spectroscopy as biomarkers of age-related loss of skeletal muscle quantity and quality. J Gerontol A Biol Sci Med Sci.

[23] Fricke H, Morse S (1925). The electrical resistance and capacity of blood for frequencies between 800 and 4½ million cycles. J Gen Physiol.

[24] Kapur K, Nagy JA, Taylor RS, Sanchez B, Rutkove SB (2018). Estimating myofiber size with electrical impedance myography: a study in amyotrophic lateral sclerosis MICE: EIM estimates myofiber size. Muscle Nerve.

[25] Kushner RF, Gudivaka R, Schoeller DA (1996). Clinical characteristics influencing bioelectrical impedance analysis measurements. Am J Clin Nutr.

[26] Berger J, Bunout D, Barrera G, de la Maza MP, Henriquez S, Leiva L, Hirsch S (2015). Rectus femoris (RF) ultrasound for the assessment of muscle mass in older people. Arch Gerontol Geriatr.

[27] Sato H, Kuniyasu K, Kobara K, Okada Y, Kawashima T, Shinonaga A, Yamamoto S, Yasunaga M, Hanayama K (2018). Verification of the accuracy of measuring the muscle cross- sectional area and muscle intensity of the rectus femoris using ultrasonography. Jpn J Compr Rehabil Sci.

[28] Kanda Y (2013). Investigation of the freely available easy-to-use software ‘EZR’’ for medical statistics’. Bone Marrow Transplant.

[29] Prado CM, Purcell SA, Alish C, Pereira SL, Deutz NE, Heyland DK, Goodpaster BH, Tappenden KA, Heymsfield SB (2018). Implications of low muscle mass across the continuum of care: a narrative review. Ann Med.

[30] Dos Santos L, Cyrino ES, Antunes M, Santos DA, Sardinha LB (2016). Changes in phase angle and body composition induced by resistance training in older women. Eur J Clin Nutr.

[31] Khalil SF, Mohktar MS, Ibrahim F (2014). The theory and fundamentals of bioimpedance analysis in clinical status monitoring and diagnosis of diseases. Sensors (Basel).

[32] Kyle UG, Bosaeus I, De Lorenzo AD, Deurenberg P, Elia M, Gómez JM, Heitmann BL, Kent-Smith L, Melchior JC, Pirlich M, Scharfetter H, Schols AM, Pichard C, Composition of the ESPEN Working Group (2004). Bioelectrical impedance analysis–part I: review of principles and methods. Clin Nutr.

[33] Taniguchi M, Yamada Y, Fukumoto Y, Sawano S, Minami S, Ikezoe T, Watanabe Y, Kimura M, Ichihashi N (2017). Increase in echo intensity and extracellular-to-intracellular water ratio is independently associated with muscle weakness in elderly women. Eur J Appl Physiol.

[34] Kapur K, Taylor RS, Qi K, Nagy JA, Li J, Sanchez B, Rutkove SB (2018). Predicting myofiber size with electrical impedance myography: a study in immature mice: EIM can predict myofiber size. Muscle Nerve.

[35] Yamada Y, Schoeller DA, Nakamura E, Morimoto T, Kimura M, Oda S (2010). Extracellular water may mask actual muscle atrophy during aging. J Gerontol A Biol Sci Med Sci.

[36] Yoshida T, Yamada Y, Tanaka F, Yamagishi T, Shibata S, Kawakami Y (2018). Intracellular-to-total water ratio explains the variability of muscle strength dependence on the size of the lower leg in the elderly. Exp Gerontol.

[37] Bourgeois B, Fan B, Johannsen N, Gonzalez MC, Ng BK, Sommer MJ, Shepherd JA, Heymsfield SB (2019). Improved strength prediction combining clinically available measures of skeletal muscle mass and quality. Journal of Cachexia, Sarcopenia and Muscle.

[38] Addison O, Marcus RL, Lastayo PC, Ryan AS (2014). Intermuscular fat: a review of the consequences and causes. Int J Endocrinol.

[39] Martins PC, Moraes MS, Silva DAS (2020). Cell integrity indicators assessed by bioelectrical impedance: a systematic review of studies involving athletes. J Bodyw Mov Ther.

[40] Seino S, Shinkai S, Iijima K, Obuchi S, Fujiwara Y, Yoshida H, Kawai H, Nishi M, Murayama H, Taniguchi Y, Amano H, Takahashi R (2015). Reference values and age differences in body composition of community-dwelling older japanese men and women: a pooled analysis of Four Cohort Studies. PLoS ONE.

[41] Ward LC (2019). Bioelectrical impedance analysis for body composition assessment: reflections on accuracy, clinical utility, and standardization. Eur J Clin Nutr.

